# End of treatment cone-beam computed tomography (CBCT) is predictive of radiation response and overall survival in oropharyngeal squamous cell carcinoma

**DOI:** 10.1186/s13014-021-01871-w

**Published:** 2021-08-09

**Authors:** Whitney Sumner, Sangwoo S. Kim, Lucas Vitzthum, Kevin Moore, Todd Atwood, James Murphy, Sayuri Miyauchi, Joseph A. Califano, Loren K. Mell, Arno J. Mundt, Andrew B. Sharabi

**Affiliations:** 1 Department of Radiation Medicine and Applied Sciences, San Diego Moores Cancer Center, University of California, 3855 Health Sciences Drive, MC 0843, La Jolla, CA 92093 USA; 2 School of Medicine, University of California, San Diego, La Jolla, CA USA; 3 Moores Cancer Center, University of California, San Diego, La Jolla, CA USA; 4 Department of Surgery, Division of Otolaryngology, University of California, San Diego, La Jolla, CA USA

**Keywords:** Head and neck squamous cell carcinoma, Head and neck cancer, Radiotherapy, Radiation, Cone beam CT, CBCT

## Abstract

**Background:**

Image guidance in radiation oncology has resulted in significant improvements in the accuracy and precision of radiation therapy (RT). Recently, the resolution and quality of cone beam computed tomography (CBCT) for image guidance has increased so that tumor masses and lymph nodes are readily detectable and measurable. During treatment of head and neck squamous cell carcinoma (HNSCC), on-board CBCT setup imaging is routinely obtained; however, this CBCT imaging data is not utilized to predict patient outcomes. Here, we analyzed whether changes in CBCT measurements obtained *during* a course of radiation therapy correlate with responses on routine 3-month follow-up diagnostic imaging and overall survival (OS).

**Materials/methods:**

Patients with oropharyngeal primary tumors who received radiation therapy between 2015 and 2018 were included. Anatomical measurements were collected of largest nodal conglomerate (LNC) at CT simulation, end of radiation treatment (EOT CBCT), and routine 3-month post-RT imaging. At each timepoint anteroposterior (AP), mediolateral (ML) and craniocaudal (CC) measurements were obtained and used to create a 2-dimensional (2D) maximum.

**Results:**

CBCT data from 64 node positive patients were analyzed. The largest nodal 2D maximum and CC measurements on EOT CBCT showed a statistically significant correlation with complete response on 3-month post-RT imaging (r = 0.313, p = 0.02 and r = 0.318, p = 0.02, respectively). Furthermore, patients who experienced a 30% or greater reduction in the CC dimension had improved OS (Binary Chi-Square HR 4.85, p = 0.028).

**Conclusion:**

Decreased size of pathologic lymph nodes measured using CBCT setup imaging during a radiation course correlates with long term therapeutic response and overall survival of HNSCC patients. These results indicate that CBCT setup imaging may have utility as an early predictor of treatment response in oropharyngeal HNSCC.

**Supplementary Information:**

The online version contains supplementary material available at 10.1186/s13014-021-01871-w.

## Introduction

Head and Neck Squamous Cell Carcinoma is a significant health issue with more than 600,000 new cases of HNSCC diagnosed worldwide and more than 60,000 new cases per year in the United States [1]. Radiation therapy (RT) plays an integral role in the curative treatment of HNSCC and radiation with concurrent chemotherapy remains the standard of care for locally advanced oropharyngeal HNSCC [2]. Despite advances in RT the survival rate for recurrent HNSCC remains low and treatment is associated with significant long-term toxicity [3, 4]. Thus, additional strategies to personalize RT or tailor concurrent or adjuvant treatment are needed.

One of the major advances in RT over the past decade is the use of advanced imaging techniques and widespread adoption of image guided radiation therapy. Image guidance is a critical tool in radiation oncology which improves the accuracy and precision of radiation delivery. Image guidance can include plain film kV imaging prior to treatment, cone beam computed tomography (CBCT), or even magnetic resonance imaging (MRI) during treatment. The improved accuracy and precision with image guided RT also permits smaller setup error margins and decreased treatment field sizes which can reduce toxicity. During treatment of HNSCC, CBCT imaging is commonly obtained daily or weekly for setup verification and to monitor for changes in patient anatomy due to tumor response or weight loss. Over the past decade, innovations including Varian’s Iterative CBCT have tremendously improved the resolution and quality of cone beam computed tomography (CBCT) such that discrete masses and lymph node conglomerates are readily discernable [5–7]. However, this large dataset of CT information has not been routinely used to monitor treatment responses largely because of the low resolution of CBCT in the past.

Interestingly, during a 7-week course of chemoradiation some patients have rapid complete clinical responses while other patients have little to no clinical response. Previous literature has documented measurable changes in disease volume over the course of radiotherapy for HNSCC [8, 9]. Sanguinetti G., et al., tested a nodal response model based on nodal density on the simulation scan with categorization of patients by the degree of shrinkage or growth throughout the treatment period. The volume and density of nodal disease was correlated to response on post-treatment imaging, although there was no analysis of impact on overall survival [9]. Similarly, Rosen et al., described significant changes in nodal volume during radiation therapy for head and neck cancer, particularly after the fourth week of therapy, but these were not correlated to treatment response or outcomes [10]. In head and neck cancer, CBCT has been utilized to measure volumetric parotid changes with resultant prediction of chronic xerostomia [10]. Additional studies have evaluated factors including the diameter change of the primary lesion and volumetric changes in nodal size utilizing CBCT but did not correlate these findings to oncologic outcomes [11, 12]. To date, despite this readily available source of CT data, studies have not assessed whether these change in lymph node measurements can predict treatment outcomes and survival.

The current gold standard to evaluate radiation treatment responses is CT and/or positron emission tomography (PET) imaging obtained several months post-treatment. PET/CT is a suitable method of disease evaluation with a negative predictive value > 90%, although false positive rates can exceed 50% [13–18]. Furthermore, in order to allow for post-treatment inflammation to subside the PET/CT often occurs 3–4 months after completing therapy which is quite a long time after treatment and off therapy, especially in cases of residual or progressive disease.

Here, we assessed whether changes in CBCT measurements obtained *during* the course of radiation treatment could be used as an early surrogate marker of treatment responses. We correlated responses on CBCT imaging with responses on 3-month routine diagnostic imaging and overall survival (OS) in patients with oropharyngeal HNSCC.

## Methods

### Data source and patient selection

We identified 64 patients with squamous cell carcinoma of the oropharynx diagnosed between 2016 and 2018 with available follow up and treatment outcomes who received curative-intent radiation therapy at the University of California, San Diego. Patients must have completed definitive therapy with radiation with or without chemotherapy. Patients were excluded if they received post-operative radiotherapy. Patients were considered post-operative if they had received any of the following: subtotal resection, gross total resection, or neck dissection (ipsilateral or bilateral). Additionally, patients were excluded if the course of radiotherapy was prolonged by greater than one week.

### Patient demographics and treatment variables

All patients received external beam radiation therapy with intensity modulated radiation therapy (IMRT) to a minimum dose of 60 Gray (Gy) (range 60–73 Gy, median 70 Gy, standard deviation (SD) 2.56 Gy) using 2.0–2.12 Gy/fraction. The median number of radiation treatments was 35 (range 30–35) over an average of 48 days. The average patient age was 61 at the time of diagnosis. T-Classification and N-classification were collected according to the American Joint Committee on Cancer (AJCC) classification 8th edition. Fifty-seven patients received concurrent systemic therapy, while 7 patients received radiation alone. Of the patients who received systemic therapy, 51 (89.5%) received cisplatin-based chemotherapy. A total of seven patients received cisplatin combined with pembrolizumab. The remaining six patients receiving either cetuximab, carboplatin/paclitaxel or pembrolizumab.

### Definition of imaging measurements

**Fig. 1:**
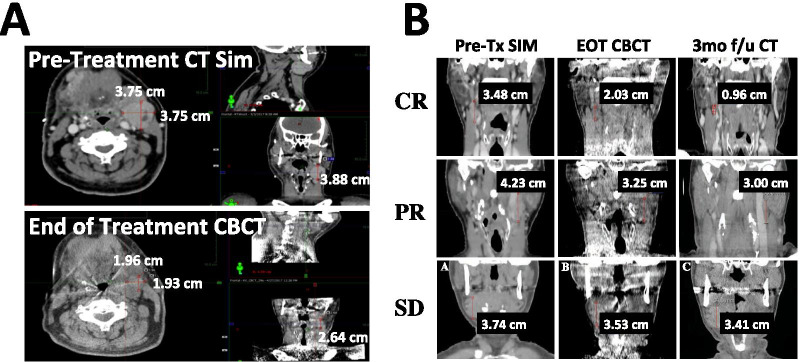
Lymph node response assessment via Cone Beam CT (CBCT) daily setup imaging versus diagnostic CT. **A** Representative patient images demonstrating CC, ML and AP measurements at CT simulation (upper panel) and End of Treatment CBCT (lower panel). **B** Representative patient images demonstrating Complete Response (upper), Partial Response (middle) and Stable Disease (lower) with CC measurements taken at Pre-Treatment CT simulation, End of Treatment CBCT and 3 month post-RT

The largest nodal conglomerate (LNC) was evaluated for measurement due to distinct fat planes and tissue heterogeneity in the cervical chain. Three timepoints were selected for measurements: (1) CT Simulation Scan (CT Sim), (2) End of Treatment (EOT) CBCT and (3) Post-Treatment Follow-up Imaging (Post-RT). The largest nodal conglomerate was identified on the CT Sim. Measurements were then collected of the LNC including anteroposterior (AP), mediolateral (ML) and craniocaudal (CC) dimensions. The AP and ML measurements were obtained from multiple axial slices of the LNC. The axial slice that resulted in the largest product of AP and ML measurements was recorded as the 2D maximum (2D). These measurements were then collected from the EOT CBCT and Post-RT with matching of bony landmarks and soft tissue structures to ensure consistency among measurements. All measurements were collected by a single operator to ensure consistency. An example of the measurement technique is shown in Fig. 1.

The EOT CBCT measurements were reported as a percent change in size relative to CT Sim. The Post-RT measurements were reported as response based on RECIST 1.1 criteria.

### Statistical analysis

Statistical analyses were performed using SPSS V23.0 (SPSS Inc., Chicago, IL) and R, version 3.5.1(R; Vienna, Austria). Median follow up was calculated using the reverse Kaplan–Meier method (KM) [19]. Correlation of percent change in size seen on EOT CBCT to Post-RT response was evaluated utilizing Pearson correlation. The Post-RT response was dichotomized to complete response (CR, ‘Responders’) or less than CR (‘Non-responders’), to include partial response (PR), stable disease (SD) or progressive disease (PD) thus creating a point biserial correlation coefficient. Logistic regression was utilized to derive the relationship between the percent change in size on EOT CBCT and response on Post-RT imaging. OS was examined using the KM method. Univariate survival analysis (UVA) was performed with the log-rank test and unadjusted Cox proportional hazards models to estimate hazard ratios (HR), with HR > 1 corresponding to worse OS and progression free survival (PFS).

## Results

A total of 64 patients were included with a median follow up of 1.6 years (range 0.7–3.7) and patient and treatment characteristics are presented in Table 1. The median patient age at diagnosis was 59.5 years (range 39–87). Forty-two patients (65.6%) had stage I-II disease. The vast majority of patients were p16-positive, n = 58 (90.7%). A total of 57 patients received systemic therapy with regimens including cisplatin (n = 47), cisplatin plus pembrolizumab, cetuximab and carboplatin/paclitaxel. A total of 8 patients (12.5%) experienced a locoregional failure and 4 patients (6.3%) experienced distant metastasis including liver, lung and bone. Average time to failure was 0.56 years.

**Table 1:** Patient characteristics

	N = 64 (%)
Age	
≤ 60	34 (53.1)
> 60	30 (46.9)
Gender	
Male	55 (85.9)
Female	9 (14.1)
Smoking	
< 10 pk yrs	34 (53.1)
≥ 10 pk yrs	30 (46.9)
T stage	
1	17 (26.6)
2	22 (34.3)
3	12 (18.8)
4	13 (20.3)
N stage	
1	33 (51.6)
2	30 (46.9)
3	1 (1.6)
Overall stage	
I	25 (39.1)
II	17 (26.6)
III	16 (25.0)
IV	5 (7.8)
P16 status	
Positive	58 (90.1)
Negative	6 (9.4)
Concurrent chemotherapy	
Yes	57 (89.1)
No	7 (10.1)
Subsite	
BoT	30 (46.9)
Tonsil	29 (45.3)
Soft palate	3 (4.7)
Pharyngeal wall	2 (3.2)

### Characteristics of complete responders and non-complete responders

On post-therapy imaging including PET/CT or CT Soft Tissue Neck, a total of 40 patients had experienced a CR, while 19 patients experienced PR and 5 patients had SD. There were no statistically significant differences in any patient characteristics between Complete responders and Non-Complete responders. Of the patients who experienced a CR, 75% had stage I-II disease as compared to 50% in non-responders (p = 0.19). Approximately 90% of both responders and non-responders were p16 positive. Representative examples of CR, PR and SD are shown in Fig. 1, and patient characteristics according to treatment response are shown in Table 2.

**Table 2:** Patient characteristics by treatment response

	Complete responders, n = 40 (62.5%)	Non-complete responders, n = 24 (37.5%)	
Age			
≤ 60	24 (60.0)	10 (41.7)	
> 60	16 (40.0)	14 (58.3)	p = 0.21
Gender			
Male	34 (85.0)	21 (87.5)	
Female	6 (15.0)	3 (12.5)	p = 0.32
Smoking			
< 10 pk yrs	20 (50.0)	14 (58.3)	
≥ 10 pk yrs	20 (50.0)	10 (41.7)	p = 0.21
T stage			
1	9 (22.5)	8 (33.3)	
2	16 (40.0)	6 (25.0)	
3	8 (20.0)	4 (16.7)	
4	7 (17.5)	6 (25.0)	p = 0.32
N stage			
1	23 (57.5)	10 (41.7)	
2	17 (42.5)	13 (54.2)	
3	0 (0.0)	1 (4.2)	p = 0.44
Overall stage			
I	16 (40.0)	9 (37.5)	
II	14 (35.0)	3 (12.5)	
III	8 (20.0)	8 (33.3)	
IV	2 (5.0)	3 (12.5)	p = 0.19
P16 status			
Positive	36 (90.0)	22 (91.7)	
Negative	4 (10.0)	2 (8.3)	p = 0.92
Concurrent chemotherapy			
Yes	36 (90.0)	21 (87.5)	
No	4 (10.0)	3 (12.5)	p = 0.16
Subsite			
BoT	14 (35.0)	16 (66.7)	
Tonsil	22 (55.0)	7 (29.2)	
Soft palate	3 (7.5)	0 (0.0)	
Pharyngeal wall	1 (2.5)	1 (4.2)	p = 0.31

Distribution of relevant patient characteristics between responders and non-responders with corresponding chi-square test

### Initial response to therapy

The median percent change in CC and 2D measurements of the LNC from CT Sim to EOT CBCT was 28.8% and 56.9% respectively in Complete responders compared to 7.7% and 38.2%, respectively, in Non-Complete responders (Table 3). The percent change in both the 2D and CC measurements from CT Sim to EOT correlated to response on Post-RT imaging with r = 0.313, p = 0.02 and r = 0.318, p = 0.02, respectively.

**Table 3:** Intra-treatment change in nodal size by treatment response

	Complete responders	Non-complete responders
CC percent change from CT Sim to EOT CBCT		
Median	28.8%	7.7%
Range	2.5–75.3	0.4–44.4
2D percent change from CT Sim to EOT CBCT		
Median	56.9%	38.2%
Range	1.8–82.9	7.6–73.8
Volumetric percent change from CT Sim to EOT CBCT		
Median	65.3%	48.8%
Range	10.9–95.6	9.8–70.0

Percent change in craniocaudal (CC), two-dimensional (2D) and volumetric size of largest nodal deposit as measured from the time of CT simulation (CT Sim) to the end-of-treatment cone beam CT scan (EOT) reported as median and range for responders and non-responders

**Fig. 2:**
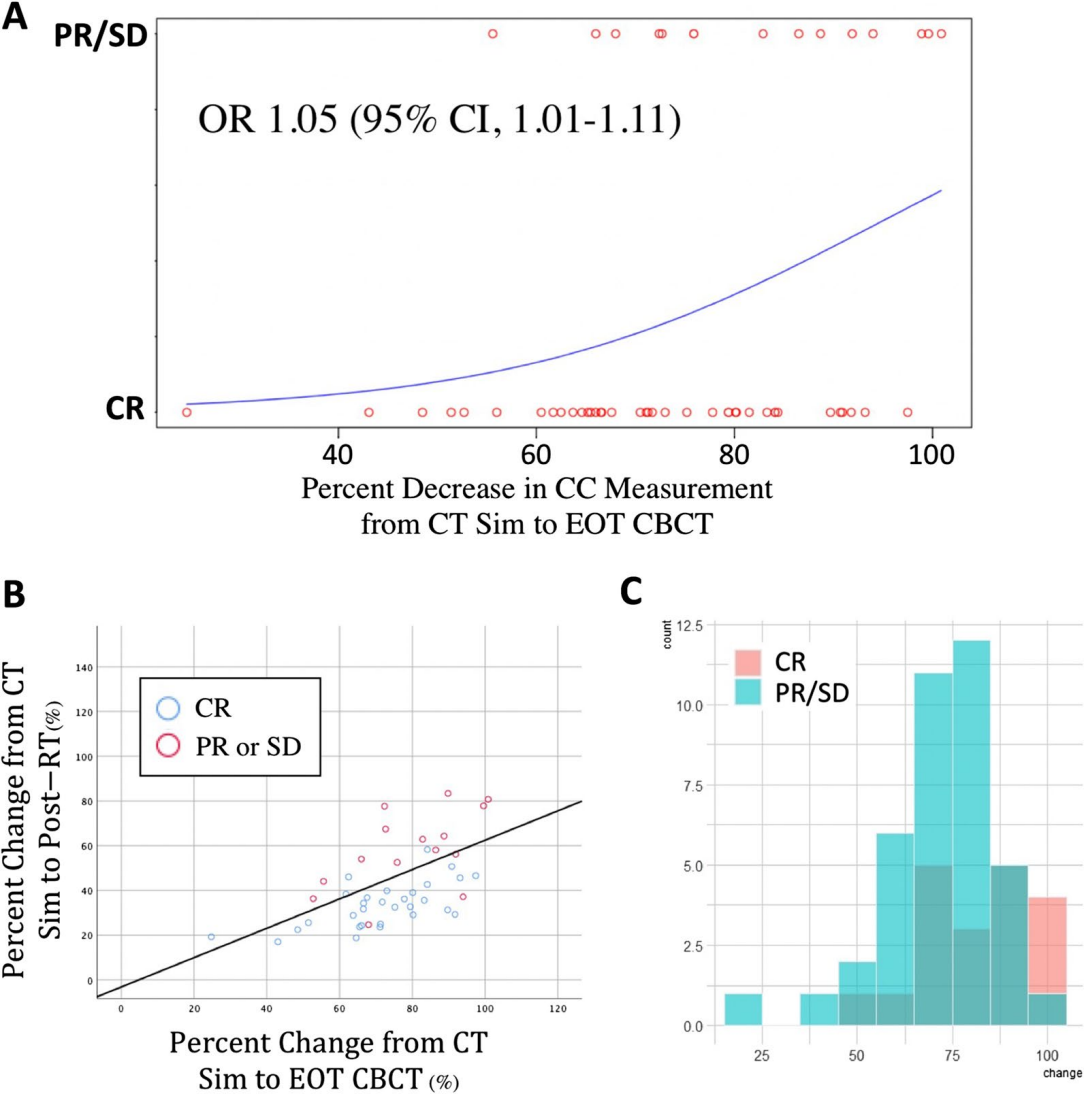
Change in size of lymph nodes at End of Radiation Treatment correlates with objective responses at 3 months. **A** Logistic regression showing the probability of response on 3-month post treatment imaging based on percent change of CC measurement from CT Sim to EOT CBCT. **B** Scatter plot demonstrating differential percent residual disease between complete responders versus patients with partial responses or stable disease. **C** Histogram demonstrating differential frequency of patients with 100% decrease at end of treatment between PR/SD versus CR. Patients were binned by each 10% change and stratified by CR compared to PR and SD

Logistics regression of the percent change in CC measurement from CT Sim to EOT CBCT based on treatment response on post-therapy imaging yielded an OR 1.05 (95% CI, 1.01–1.11) reflecting a 5% increase in CR for every 1% change from CT sim to EOT CBCT (Fig. 2). For patients who experienced $\ge$ 40% (n = 8) and $\ge$ 30% (n = 17) reduction in CC measurement from CT Sim to EOT CBCT, the rate of CR was 87% and 82% respectively. Conversely, among patients who experienced < 10% (n = 11) reduction CC measurement from CT Sim to EOT CBCT, the rate of CR was 45%. Additionally, a $\ge$ 30% reduction in CC measurement from CT Sim to EOT CBCT was significantly associated with CR on post-therapy imaging (r = 0.274, p = 0.039) which supports our hypothesis that EOT CBCT may have utility as an early indicator of treatment response.

### Survival analysis

**Fig. 3:**
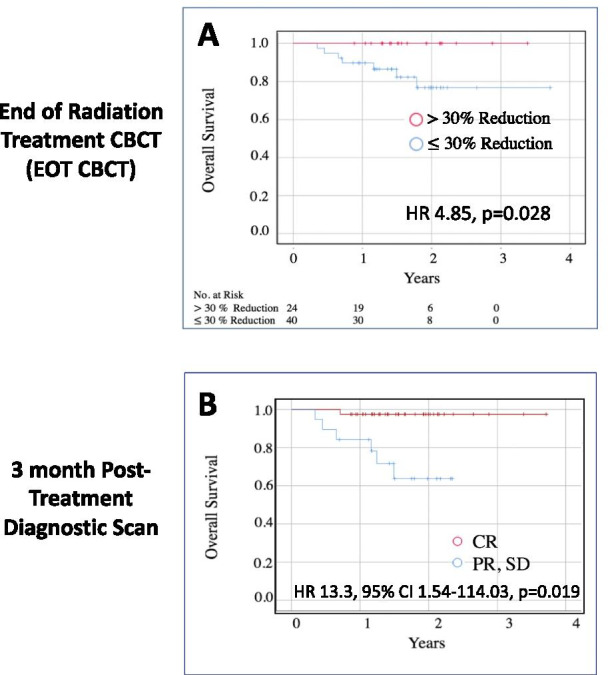
Reduced lymph node size at end of radiation treatment correlates with overall survival. **A** Unadjusted Kaplan–Meier curve demonstrating overall survival for patients who experienced > 30% (red) and ≤ 30% reduction (blue) in the CC measurement from CT simulation to EOT CBCT (Binary Chi-Square HR 4.85, p = 0.028; continuous variable HR 1.07, 95% CI 1.02–1.15, p = 0.045). Patients with a > 30% decrease in lymph node size at end of treatment demonstrated improved overall survival. **B** Unadjusted Kaplan–Meier curve demonstrating overall survival for patients who experienced a CR (blue) and less than CR (PR, SD) (red) on 3-month post-treatment imaging

Out of 64 patients a total of five patients experienced local failure with two of these patients additionally developing distant metastases in the lung and chest wall, respectively. A single patient developed isolated liver metastases. Two-year OS was 100% in patients who had a $\ge$ 30% reduction in the CC measurement compared to 78% for those with $<$ 30% reduction ( Binary Chi-Square HR 4.85, p = 0.028; continuous HR 1.07, 95% CI 1.02–1.15, p = 0.045) (Fig. 3A). Two-year PFS in these cohorts with $\ge$ 30% reduction versus $<$ 30% reduction was 100% and 86%, respectively (HR 1.06, 95% CI 0.99–1.14; p = 0.11). Analysis of PFS and OS based on 2D measurements of the LNC from CT Sim to EOT CBCT did not reach statistical significance (HR 1.03, 95% CI 0.996–1.07 and HR 1.07, 95% CI 0.99–1.05, p = 0.28, respectively). In order to compare the prognostic capability of a ≥ 30% reduction in the EOT CBCT metric to the standard 3 month post-treatment diagnostic scan, we analyzed overall survival and progression free survival in patients that manifested a CR versus non-CR on diagnostic imaging. Two-year OS in patients that had a CR versus non-CR on post-treatment diagnostic imaging was 97% and 67%, respectively (Fig. 3B). Two-year PFS in patients that had a CR versus non-CR on post-treatment diagnostic imaging was 100% and 56%, respectively. Kaplan–Meier curves for PFS are shown in Additional file 1: Figure 1. Importantly, the 2 year overall survival in patients with a ≥ 30% reduction in the EOT CBCT CC measurement was almost identical to the 2 year overall survival estimates predicted in patients that demonstrated a CR on post-therapy imaging, namely 100% and 97% respectively (Fig. 3). These findings suggest that EOT CBCT imaging data may have utility as an early prognostic indicator of treatment response.

## Discussion

Across the United States radiation oncologists will treat nearly 50,000 HNSCC patients with radiation therapy each year. During treatment, weekly or daily CBCT setup imaging data is acquired for each patient and the quality of this setup imaging continues to improve. However, this setup imaging data is not currently used for prognostication or therapeutic guidance. Here we analyzed CBCT imaging data from 64 patients and determined that changes in pathologically enlarged LN correlated with objective responses and patients with a ≥ 30% decrease in pathologically enlarged LN on the last day of radiation treatment had improved overall survival.

This study contributes to a growing area of research into the utilization of radiation image guidance data to assess tumor response and outcomes. Previous studies have evaluated toxicity outcomes and tumor regression during the treatment period, though without correlation to overall survival [10, 11]. Interestingly, a prospective institutional study from Taipei Medical University utilized PET/CT during the 5th week of definitive chemoradiation and reported a higher local recurrence for patients with higher mid-treatment SUVm value [20]. While insightful, PET/CT imaging during or immediately after a course of radiation therapy can be problematic to interpret due to edema, mucositis, and other inflammatory changes caused by radiation or chemotherapy itself. The current standard of care used to determine therapeutic response is PET/CT or CT at 12–16 weeks post-therapy in conjunction with physical exam and nasopharyngolaryngoscopy. Post-RT imaging is well-correlated to treatment outcomes with findings ultimately guiding management such as neck dissection for patients with residual FDG avid neck disease [18]. Importantly PET imaging has a high negative predictive value of 90%, which, as demonstrated in the PET-NECK trial, can spare the patients the morbidity of neck dissection without compromising treatment outcomes [21, 22]. Nevertheless, 3–4 months is a substantial time to wait to determine therapeutic response and earlier assessments could help to inform patients and potentially guide adjuvant therapy.

When we analyzed demographics from our 64 patients with oropharyngeal HNSCC, we observed the vast majority (90%) were p16+, consistent with rising rates of HPV positivity as well as low rates of smoking in our region (Table 1). While CBCT imaging was originally designed to help setup patients using soft tissue as well as boney anatomy, improved algorithms and detection arrays now permit distinct identification of tumor masses and LNs. Nevertheless, for oropharyngeal HNSCC we were unable to obtain precise measurements of the primary tumor site using CBCT due to the lack of contrast between the primary tumor and surrounding normal mucosal and soft tissues. However, we were able to obtain accurate and robust measurements of LN and nodal conglomerates due to contrast with surrounding fat planes and more uniform shapes and used this for our analyses. We observed that changes in LN CC dimension at the end of treatment CBCT had the strongest correlation with complete response rate with a median change of 28.8% from CT simulation to end of treatment in patients with CR versus only 7.7% in non-complete responders. These data indicate that patients with rapid clinical responses to radiation are more likely to have complete responses at 3-month post-treatment PET/CT.

Ultimately it would be beneficial to have an early predictor of treatment response which could help to guide management such as de-escalation or treatment intensification. This would be especially beneficial in light of multiple ongoing Phase III studies using checkpoint blockade immunotherapy either concurrently during chemoradiation or adjuvantly after completion of chemoradiation [23–25]. However, because outcomes are relatively favorable for intermediate risk HPV + HNSCC patients, additional escalation of treatment, such as immunotherapy after completion of chemoradiation, may be unnecessary in certain patients. When we analyzed survival in our patients, we found that those patients who manifested a ≥ 30% reduction in LN CC measurement from CT simulation to EOT CBCT had significantly improved overall survival (Fig. 3). Taken together, this finding indicates that CBCT at the end of treatment may have utility as an imaging modality for early identification of complete responders and stratification of patients with significantly improved overall survival.

Further prospective studies would be needed to determine whether CBCT patients with decreased size of pathologic LN at the end of radiation treatment have improved overall survival. In the Phase III Pacific study consolidative anti-PD-L1 immunotherapy was administered to patients between 1–42 days after completion of chemoradiation for lung cancer. Given that the standard post-treatment imaging for HNSCC is routinely obtained around 90 days post-treatment, this creates a significant delay in the initiation of a potentially beneficial therapy. Due to the lack of robust literature correlating intra-treatment imaging to disease outcomes, there are several ongoing trials evaluating de-escalation of therapy by utilizing PET or CT imaging at designated time points during the treatment window including Memorial Sloan Kettering’s Major Radiation Reduction for HPV + OPSCC (NCT03323463) and the Quarterback Trial (NCT01706939) at Mount Sinai. Future prospective validation of intra-treatment imaging as a predictor of treatment response in HNSCC could provide additional prognostic indicators for therapeutic clinical trials.

There are multiple limitations of this retrospective study. The use of CBCT imaging is a novel technique and standards for measurements of tumors and LN have not been established. CBCT is also prone to artifacts due to the wide incident cone-beam field and reconstruction and filtering algorithms required. Additionally, there are patients who have a partial response or stable disease at end of treatment that go on to have a durable complete response. Nevertheless, we believe that these results identify end of treatment CBCT as a novel imaging tool to identify rapid responders who are likely to manifest durable complete responses with improved outcomes. It is also important to note that approximately 90% of our cohort was comprised of patients with HPV-associated oropharyngeal HNSCC, which limits interpretation of these findings for HPV- oropharyngeal HNSCC.

Ultimately, the next steps for precision radiation medicine involve correlation of molecular and radiomic findings with treatment outcomes to guide therapeutic recommendations. From RTOG 0129, we have long understood that HPV-associated HNSCC is a distinct entity from HPV-negative malignancies [26]. Recent studies have identified molecular and genomic subtypes that correlate with outcomes of patients with HPV associated and HPV negative Head and Neck cancers [27–30]. However, additional work is needed to validate molecular profiles and genomic alterations which are predictive of radiation responses in HNSCC.

## Conclusion

Novel therapeutic approaches and combinations of surgery, radiation, and systemic therapies continued to be tested in HNSCC. We strongly believe that the ability to correlate imaging data to treatment outcomes, and ultimately incorporating molecular profiling, is certain to serve a pivotal role in the era of personalized medicine. To our knowledge, this is the first study to correlate these intra-treatment imaging findings to treatment response and survival outcomes. Furthermore, we utilized a technique that can be readily applied in clinical practice. Future studies are needed to expand this dataset and validate the predictive capacity of intra-treatment imaging modalities on long term patient outcomes.

## Supplementary Information

**Additional file 1: Figure 1.** Complete response on post-treatment imaging results in improved in progression-free survival. **A** Unadjusted Kaplan–Meier curve demonstrating progression-free survival for patients who experienced a CR (blue) and less than CR (PR, SD) (red) on 3-month post-treatment imaging.

## Data Availability

The datasets used and/or analyzed during the current study are available from the corresponding author on reasonable request.

## Abbreviations

RT: Radiation therapy
CBCT: Cone beam computed tomography
HNSCC: Head and neck squamous cell carcinoma
OS: Overall survival
LNC: Largest nodal conglomerate
EOT: End of treatment
AP: Anteroposterior
ML: Mediolateral
CC: Craniocaudal
MRI: Magnetic resonance imaging
PET: Positron emission tomography
IMRT: Intensity modulated radiation therapy
AJCC: American Joint Committee on Cancer
Post-RT: Post-treatment follow-up imaging
KM: Kaplan–Meier method
CR: Complete responder
PR: Partial responder
SD: Stable disease
PD: Progressive disease
UVA: Univariate survival analysis
HR: Hazard ratio
PFS: Progression free survival
