# Supplementary figures and images for: End of treatment cone-beam computed tomography (CBCT) is predictive of radiation response and overall survival in oropharyngeal squamous cell carcinoma
Source: Radiat Oncol. 2021 Aug 9;16:147. doi: 10.1186/s13014-021-01871-w (PMC8351145; doi:10.1186/s13014-021-01871-w)

A

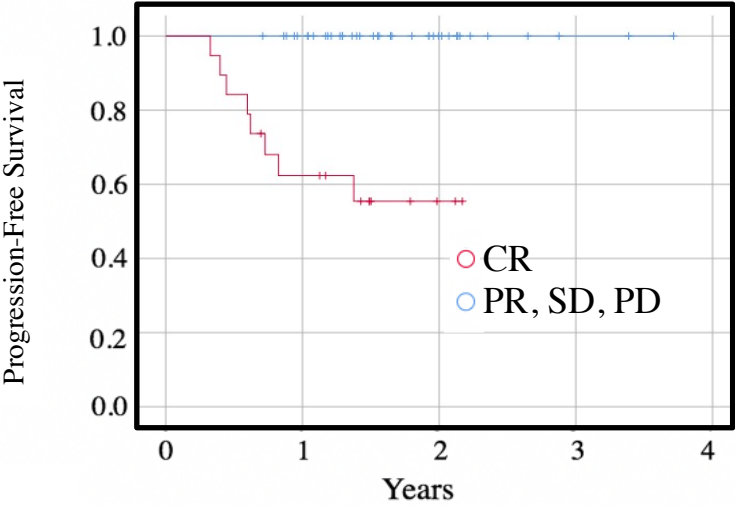

Supplement: Supplementary file 1 — Additional file 1: Figure 1. Complete response on post-treatment imaging results in improved in progression-free survival. A Unadjusted Kaplan–Meier curve demonstrating progression-free survival for patients who experienced a CR (blue) and less than CR (PR, SD) (red) on 3-month post-treatment imaging. [file 13014_2021_1871_MOESM1_ESM.pdf]
